# Tuberculous Peritonitis

**DOI:** 10.1155/S1064744997000641

**Published:** 1997

**Authors:** David E. Soper

**Affiliations:** Department of Obstetrics and Gynecology Medical University of South Carolina 171 Ashley Avenue Charleston SC USA


					Infectious Diseases in Obstetrics and Gynecology 5:360 (1997)
(C) 1998 Wiley-Liss, Inc.
Images in Infectious Diseases in Obstetrics and Gynecology
Section Editor: David E. Soper, M.D.
Tuberculous Peritonitis
David E. Soper, MD
Department of Obstetrics and Gynecology, Medical University
ofSouth Carolina, Charleston, SC
A 45-year-old Indian woman presented with a large pel-
vic mass and ascites. A preoperative diagnosis of ovarian
carcinoma was made, and exploratory laparotomy was
performed. Findings at surgery revealed multiple nod-
ules on the omentum (A), peritoneum, fallopian tubes,
and serosa of the uterus. A tubal biopsy was performed.
Tissue examination confirmed the presence of caseating
granulomas (B, C). The patient was treated for 12
months with antituberculous therapy (isonizid and ri-
fampin) and did well. (C) 1998 Wiley-Liss, Inc.
A
Images in Infectious Diseases in Obstetrics and Gynecology presents clinically important visual images that a practitioner in women's health
might encounter. If you have a high-quality color or black-and-white photograph or slide representing such an image that you would
like considered for publication, send it with a descriptive legend to David E. Soper, MD, Department of Obstetrics and Gynecology,
Medical University of South Carolina, 171 Ashley Avenue, Charleston, SC 29401-2233.
Images is made possible through an educational grant from Pfizer, Inc.
Images

				

